# Supplementary material for: MetaRibo-Seq measures translation in microbiomes
Source: Nat Commun. 2020 Jun 29;11:3268. doi: 10.1038/s41467-020-17081-z (PMC7324362; doi:10.1038/s41467-020-17081-z)
Supplement: Supplementary file 10 — Supplementary Data 7 [file 41467_2020_17081_MOESM10_ESM.zip › File2/Confidence_High_Taxonomy/97918_out.krona.html]

Javascript must be enabled to view this page.

members
magnitude
magnitudeUnassigned
count
unassigned
taxon
rank

97918\_out

2

1
superkingdom
2

1
phylum
1239

1
class
186801

order
186802
1

1392389
genus
1

1

SRS097958\_contig\_number\_10903
species
1297617

1

SRS147346\_contig\_number\_contig-100\_14857.14857
